# From Fleming to *Lifeline*: theatre, narrative and public engagement in antimicrobial stewardship

**DOI:** 10.1093/jacamr/dlag185

**Published:** 2026-08-25

**Authors:** Rasha Abdelsalam Elshenawy, Marvelle Brown

**Affiliations:** Public Health and Patient Safety Unit, Department of Medicine, Centre for Health Services and Clinical Research, School of Health, Medicine and Life Sciences, University of Hertfordshire, Hatfield, Hertfordshire AL10 9AB, UK; Public Health and Patient Safety Unit, Department of Medicine, Centre for Health Services and Clinical Research, School of Health, Medicine and Life Sciences, University of Hertfordshire, Hatfield, Hertfordshire AL10 9AB, UK

## Abstract

Antimicrobial resistance (AMR) is a defining global health threat, yet public understanding of why antibiotic stewardship matters remains limited and statistical communication alone is rarely sufficient. Arts-based interventions offer a complementary route to engagement that reaches the affective domain of learning. Lifeline, a musical staged at Southwark Playhouse Elephant in May 2026 by Charades Theatre Company, which evolved from the company’s formally evaluated predecessor production *The Mould that Changed the World*, dramatizes Sir Alexander Fleming’s discovery of penicillin alongside contemporary clinical narratives, including a patient who dies because no effective antibiotic remains, and another who survives through stewardship-supported care. Its rotating chorus of real-life clinicians, scientists and policy-makers models the One Health response that AMR demands. Drawing on the experience of taking postgraduate medicine, Master of Public Health students from the University of Hertfordshire to attend the production, this Viewpoint argues that arts-based public engagement merits a recognized, funded and evaluated place within antimicrobial stewardship education and public-health communication.

## Why narrative?

Communicating the urgency of antimicrobial resistance (AMR) to learners, patients and the public remains one of the central challenges of antimicrobial stewardship. Statistical projections of millions of deaths attributable to drug-resistant infections^1^ rarely move audiences in the way that a single patient story does. Those of us teaching antimicrobial stewardship to Master of Public Health (MPH) and other postgraduate students return repeatedly to narrative: the patient who deteriorated despite escalating therapy, the empty antibiogram, the conversation with relatives that no susceptibility report can prepare a clinician for. Narrative is how we make resistance real.

## Lifeline: two patient journeys and a one health chorus

*Lifeline*, a new musical produced by Charades Theatre Company and staged at Southwark Playhouse Elephant in May 2026, demonstrates how that pedagogy can be scaled beyond the classroom. With music and lyrics by Robin Hiley, a book by Becky Hope-Palmer and direction by Alex Howarth, and supported by its patron Professor Dame Sally Davies, UK Special Envoy on AMR, the production interweaves the historical story of Sir Alexander Fleming’s discovery of penicillin^2^ with two contrasting patient journeys. In the first, a young man dies of an infection that no available antibiotic can cure, and, in a deliberate dramatic choice, he is the partner of one of the hospital’s own doctors. His death is not the result of negligence but of a therapeutic landscape stripped bare by resistance, and the storyline’s force lies in watching a clinician confront the limits of her profession when she cannot save the person she loves most. In the second, a young patient recovers from a serious infection because effective antibiotics, preserved by stewardship, infection prevention, vaccination and a multidisciplinary team, remain available when she needs them.

The juxtaposition is not abstract; it is dramatized, sung and felt, setting in successive scenes the two outcomes that AMR delivers in real-world practice: the loss that follows when the lifeline of effective antimicrobials is broken, and the recovery that remains possible when it is preserved. The grief song that follows the bereavement, ‘The Boys Are Home’, is among the most affecting public-health writing seen on the British stage.

The Fleming storyline reinforces the argument with the original warning: in his 1945 Nobel Lecture, Fleming cautioned that subtherapeutic and inappropriate use of penicillin would not eradicate bacteria but would instead ‘educate’ them to resist, a prophecy that has proved devastatingly accurate.^2^ For an MPH learner who has studied resistance epidemiologically, the same arc takes on a different texture when sung, grieved and celebrated on stage. Importantly, *Lifeline* does not portray antibiotics as the enemy. It reaffirms them as one of the most precious tools of modern medicine, while making clear that their continued effectiveness depends on stewardship, judicious prescribing, surveillance, diagnostics and innovation, the integrated response advocated by the WHO Global Action Plan on AMR and the UK national action plan.^3,4^

**Figure 1.**
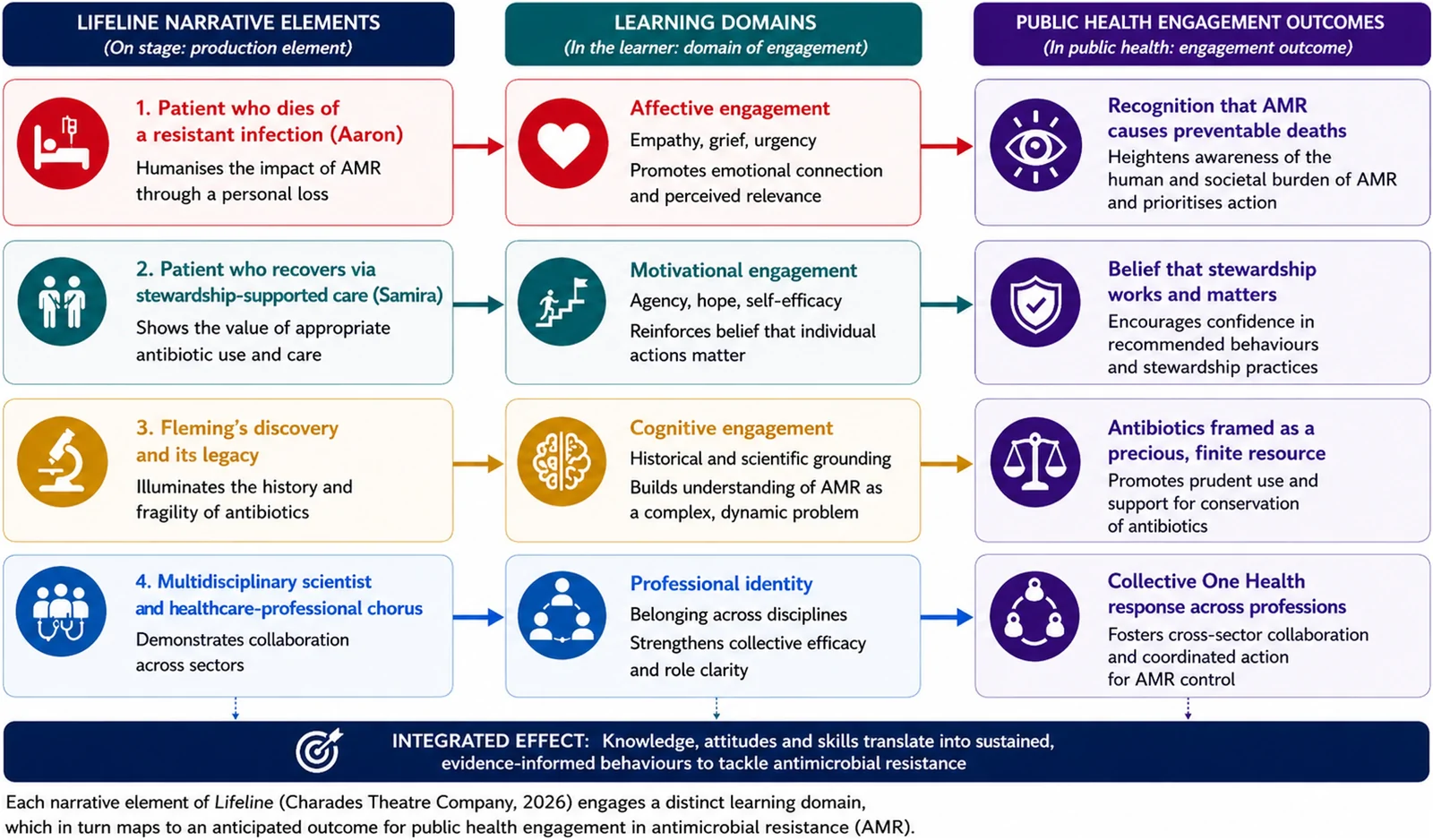
Conceptual framework linking *Lifeline* narrative elements to AMR learning and public health engagement outcomes. Conceptual framework showing how four narrative elements of Lifeline (Charades Theatre Company, 2026) map to learner engagement domains and anticipated public health engagement outcomes. Each narrative element engages a distinct learning domain, which in turn maps to an anticipated outcome for public health engagement in AMR.

A second feature of *Lifeline* distinguishes it from earlier arts-based health interventions. Each performance is supported by a rotating *Lifeline* Scientist and Healthcare Professional Chorus of real clinicians, scientists and policy-makers: paediatric ENT surgeons, GPs, infectious diseases physicians, microbiologists, nurses, veterinary epidemiologists, climate-and-AMR researchers, science communicators and industry leaders working on antibiotic research, regulation and access. The chorus is more than spectacle; it is a working tableau of the One Health, multidisciplinary response that AMR demands.^5^ The reach of this multidisciplinary model extends beyond the theatre: *Lifeline* became the first musical to perform on the floor of the United Nations General Assembly, and its UK run has been accompanied by a co-ordinated programme of stewardship-themed panel discussions, school workshops and a parliamentary gala convened by the production’s patron and the Chair of the All-Party Parliamentary Group on Antimicrobial Resistance. The visible inclusion of pharmacists and public-health professionals in this picture matters: pharmacists steward antimicrobial use through prescribing review, intravenous-to-oral switch, allergy de-labelling and patient counselling, while public-health professionals contribute surveillance, outbreak response, vaccination programmes and behaviour change communication. A musical that puts these roles on stage offers something that conventional health communication seldom achieves, a positive, collective vision of professional identity in the face of a slow-moving crisis. Together, these four narrative elements engage distinct learning domains and map onto corresponding public-health engagement outcomes (Figure 1).

## From the mould that changed the world to lifeline: an evaluated lineage

*Lifeline* did not emerge in a vacuum. It evolved from *The Mould that Changed the World*, an earlier musical by the same composer and theatre company, conceived with the infectious diseases physician Dr Meghan Perry and supported by the British Society for Antimicrobial Chemotherapy, which first told Fleming’s story as a participatory primary-school production and later as a professional show performed with a chorus of NHS clinicians and scientists. Crucially for the present argument, that predecessor has been formally evaluated, with results reported in *The Lancet* and published in full in *PLOS One*.^6,7^ In a mixed-methods study of 182 children aged 9–11 years who rehearsed and performed the musical in two UK primary schools, participants were significantly more likely to answer questions on its key messages correctly two weeks after performance than before rehearsals began (for example, knowledge that bacteria can become resistant to antibiotics, odds ratio 4.63, 95% CI 2.46–9.31), and focus groups described changed attitudes and motivation to reduce personal antibiotic use and to influence family and friends.^7^ More recently, an evaluation of *Lifeline* itself, published in *Nature Medicine*, reported that the production raised AMR awareness and inspired intention to act among its audiences.^8^ These published findings, spanning participating children, professional chorus members and adult theatre audiences, add weight to the argument that musical theatre is a credible and transferable vehicle for engaging both adults and children with the AMR message.

## Implications for AMR education and public engagement

What does this mean for those of us responsible for AMR education and public engagement? Three implications follow. First, arts-based public engagement deserves a recognized, funded place within MPH and other postgraduate curricula, not as an optional extra but as a structured learning activity with explicit outcomes around AMR awareness, professional identity and health communication. We took MPH students from the University of Hertfordshire to attend *Lifeline*; their written reflections demonstrated learning that extended beyond cognitive recall into the affective and motivational domains that traditional teaching rarely reaches.^9^ Second, stewardship programmes should consider incorporating patient narratives, both stories of loss and stories of recovery, into staff induction and continuing professional development, mirroring the dual-narrative structure that gives *Lifeline* its moral force. Third, the impact of arts-based interventions must be evaluated rigorously. The collaboration between *Lifeline* and the University of Edinburgh has already begun to yield published findings,^8^ this should now be followed by further outcome data on knowledge, attitudes and behaviour change. A central question, raised whenever arts-based engagement is proposed, is whether the learning it generates is sustained. Here the published evidence, although still limited, is encouraging: in the evaluation of *The Mould that Changed the World*, children’s correct responses on the musical’s key messages remained consistent between two weeks and six months after performance in the school followed up over that period, indicating durable knowledge gain rather than a transient effect.^7^ Longitudinal data beyond 6 months, and for adult audiences, remain scarce, a gap recognized across arts-in-health research more broadly.^9^ Future evaluations, including of *Lifeline*, should therefore incorporate repeated follow-up. Within curricula, a single theatrical encounter is most likely to be consolidated into sustained learning when it is reinforced by linked activities such as reflective assignments, seminar discussion and stewardship teaching, which is how we embedded the *Lifeline* visit within our MPH programme.

Theatre will not, on its own, solve AMR. Stewardship, surveillance, diagnostics, vaccines, equitable access to medicines and sustained policy investment are doing that work and must continue. But communication is not peripheral to that response, it is constitutive of it. As *Lifeline* reminded its audience, change does not come from waiting for a miracle; it comes from people, often in small groups, who choose to act. The closing chorus is, in the end, a public-health argument: protect the lifeline, and many more patients will live to tell their own stories. For clinicians, scientists and educators committed to that protection, supporting and learning from productions such as *Lifeline* is no longer a fringe activity. It is part of the work.

## Transparency declarations

R. A. E. is an Associate Editor of *JAC-Antimicrobial Resistance*; this manuscript has been prepared independently of that role, R. A. E. has had no access to the submission through the online system and will have no involvement in its editorial handling. R. A. E. and M. B. organized the University of Hertfordshire Master of Public Health student attendance at *Lifeline* that informs this Viewpoint, and have no financial relationship with Charades Theatre Company or with any of the production’s commercial sponsors. Both authors contributed to the conception, drafting and critical revision of this Viewpoint and approved the final version. All authors: none other to declare.
